# Interactions Between *Bacillus* Spp., *Pseudomonas* Spp. and *Cannabis sativa* Promote Plant Growth

**DOI:** 10.3389/fmicb.2021.715758

**Published:** 2021-09-20

**Authors:** Dominique Comeau, Carole Balthazar, Amy Novinscak, Nadia Bouhamdani, David L. Joly, Martin Filion

**Affiliations:** ^1^Department of Biology, University of Moncton, Moncton, NB, Canada; ^2^Research and Development Centre, Agriculture and Agri-Food Canada, Agassiz, BC, Canada; ^3^Department of Chemistry and Biochemistry, University of Moncton, Moncton, NB, Canada; ^4^Research and Development Centre, Agriculture and Agri-Food Canada, Saint-Jean-sur-Richelieu, QC, Canada

**Keywords:** cannabis, plant growth promotion, rhizosphere, *pseudomonas*, *bacillus*

## Abstract

Plant growth-promoting rhizobacteria (PGPR) deploy several mechanisms to improve plant health, growth and yield. The aim of this study was to evaluate the efficacy of two *Pseudomonas* spp. strains and three *Bacillus* spp. strains used as single treatments and in consortia to improve the yield of *Cannabis sativa* and characterize the impact of these treatments on the diversity, structure and functions of the rhizosphere microbiome. Herein, we demonstrate a significant *C. sativa* yield increase up to 70% when inoculated with three different *Pseudomonas* spp./*Bacillus* spp. consortia but not with single inoculation treatments. This growth-promoting effect was observed in two different commercial soil substrates commonly used to grow cannabis: Promix and Canna coco. Marker-based genomic analysis highlighted *Bacillus* spp. as the main modulator of the rhizosphere microbiome diversity and *Pseudomonas* spp. as being strongly associated with plant growth promotion. We describe an increase abundance of predicted PGPR metabolic pathways linked with growth-promoting interactions in *C. sativa*.

## Introduction

Plant-growth-promoting rhizobacteria (PGPR) are rhizosphere inhabitants associated with the root system that enhance the uptake of nutrients, produce beneficial phytohormones and protect against biotic and abiotic stressors, on the whole, augmenting plant growth and fitness (Cordovez et al., 2019). Advantageously modulating the rhizosphere microbiome by inoculating with known PGPR has been practiced for nearly a century and is currently rooted in sustainable agriculture (Hartmann et al., 2008; Adesemoye et al., 2009; Bhattacharyya and Jha, 2012; Gupta et al., 2015). The isolation, identification and commercialization of PGPR inocula are implemented to promote plant growth and health in a variety of commercially important crops such as wheat, rice, corn and soybean (Etesami et al., 2014; Timmusk et al., 2017; Backer et al., 2018). Similarly, the use of PGPR which have the capacity to colonize the rhizosphere and promote plant growth and yield could greatly benefit and dynamize the nascent cannabis industry (Lyu et al., 2019). The numerous products derived from *C. sativa*, namely its fibers, nutritional food ingredients and secondary metabolites used both medicinally and recreationally are valuable resources that have begun to garner scientific and economic interest (Andre et al., 2016; Dolgin, 2018). Research centered around the characterization of *C. sativa*’s microbiome, although in its infancy, has highlighted the dynamism and plasticity of the plant’s microbiome along spatiotemporal axes and soil-type dependencies as well as potential PGPR isolated from multiple cultivars (Kusari et al., 2012; Winston et al., 2014; Scott et al., 2018; Comeau et al., 2020). The flexibility and adaptability of the microbiome hint towards the amenable colonization of *C. sativa* with compatible bacterial treatments. Initial studies have demonstrated the feasibility of this approach using a consortium of PGPR (*Azospirillum brasilense*, *Gluconacetobacter diazotrophicus*, *Burkholderia ambifaria* and *Herbaspirillum seropedicae*) or previously commercialized inoculants that potentiated the growth of *C. sativa* (Conant et al., 2017; Pagnani et al., 2018). However, the mechanism by which these inoculants benefited plant yield in consortia was not thoroughly explored.

Among the phylogenetically diverse PGPR studied to date, *Pseudomonas* spp. and *Bacillus* spp. have been consequential in the development of commercial bio-formulations (Adesemoye et al., 2009; Borriss, 2011; Santoyo et al., 2012). They are common rhizosphere inhabitants, possess an array of growth-promoting features and may potentially be prospective early colonizers capable of niche pre-emption and modification (Parret et al., 2003; Bottini et al., 2004; Humphris et al., 2005; Idris et al., 2007; Beneduzi et al., 2012; El-Sayed et al., 2014; Masciarelli et al., 2014; Islam et al., 2015; Souza et al., 2015; Schreiter et al., 2018; Ansari and Ahmad, 2019; Qessaoui et al., 2019). Mechanistically, *Pseudomonas* spp. and *Bacillus* spp. have been shown to actively colonize the rhizosphere and to promote plant growth by rendering phosphorus, iron and nitrogen bio-available as well as producing beneficial phytohormones such as indole-3-acetic acid (Berg et al., 2014; Qessaoui et al., 2019; Wei et al., 2019). The biofilm formation properties of *Bacillus* spp. are influenced by multi-species interaction, such as with *Pseudomonas* spp., and are linked to increased competitiveness and colonization in the rhizosphere (Shank et al., 2011; Powers et al., 2015; Ren et al., 2015; Ansari and Ahmad, 2019). In line with the current literature, these species have the potential to be valuable PGPR in the cultivation of *C. sativa* (Lyu et al., 2019).

In the present work, *Pseudomonas* spp. and *Bacillus* spp. isolates obtained from Canadian soils have been utilized to assess their ability to promote the growth of *C. sativa* when inoculated alone or in combination treatments in different commercial soil substrates commonly used to grow cannabis. To go beyond classical studies, the long-term effect of these treatments on the diversity, structure and functions of the microbiome have also been evaluated using Illumina marker gene sequencing of bacteria (16S rRNA gene) coupled to the QIIME2 pipeline for analysis. We demonstrate that single treatments do not stimulate growth promotion under the conditions tested, but combinatorial treatments have a significant positive effect on plant growth. The results obtained suggest that *Bacillus* spp. modulates the rhizosphere microbiome of *C. sativa* and enables the colonization of *Pseudomonas* spp., complementing plant growth independently of substrate type, hence demonstrating an interaction between *Bacillus* spp., *Pseudomonas* spp. and *C. sativa*. Furthermore, marker-based pathway predictions highlighted known PGPR metabolic pathways related to *C. sativa* plant growth promotion.

## Materials and Methods

### Bacterial and Plant Culture

#### *Pseudomonas* Spp. and *Bacillus* Spp.

*Pseudomonas fluorescens* LBUM223, *Pseudomonas protegens* LBUM825, *Bacillus velezensis* LBUM279, *Bacillus subtilis* LBUM979 and *Bacillus siamensis* LBUM1082 were previously isolated in our laboratory from the rhizosphere of *Fragaria ananassa*, i.e. strawberry plants (Bouctouche, NB, Canada) and either maintained in TSB media (Sigma-Aldrich) at 25°C/120rpm (*Pseudomonas*) or at 37°C/120rpm (*Bacillus*) for experiments and stocked at −80°C. These bacteria isolated from strawberry plants were beneficial to plant growth (data not shown), and as a beneficial bacterium from one plant could be beneficial to another, we used these bacteria to survey their efficacy in *C. sativa* (Smith et al., 2015; Lyu et al., 2019). For plant growth promotion experiments, bacterial cultures were diluted in water to a final concentration of 10^8^CFU/ml.

#### Cannabis sativa

The Anka cultivar was seeded in starting trays containing either coconut-based medium supplemented with commercial nutrient (Canna coco substrate and AB solution; Toronto, Canada) or a 1:4 mixture of vermiculite and Promix substrate (Premier Tech, Rivière-du-Loup, Canada). Canna coco (Canna coco, Toronto, Canada) is made up of coconut husks and Promix (Promix, Rivière-du-Loup, Canada) is a mix of peat moss, peat humus and perlite; both substrates were chosen because they are commonly used in the commercial growth of cannabis. After a week, the plantlets were transplanted to larger 1-l pots containing 800ml of either Canna coco or Promix substrate.

### Plant Growth Promotion Experiments

The plantlets were simultaneously inoculated with the different bacterial treatments at the time of transplantation. Plants were inoculated at the base of the stem with water (20ml control treatment), a single bacterial species (10ml of *Pseudomonas* spp. or *Bacillus* spp. + 10ml of water) or a combination of 2 species (10ml *Pseudomonas* spp. + 10ml *Bacillus* spp.) for a total of 10 different treatments (Control, LBUM223, LBUM825, LBUM979, LBUM1082, LBUM279, LBUM223/979, LBUM223/1082, LBUM825/279 and LBUM825/979). In order to keep the number of treatments to a reasonable size, some combinations not listed above were omitted as preliminary experiments indicated that these combinations did not yield plant growth-promoting effects (data not shown). Two additional controls were also prepared where plants were inoculated with double doses of *Pseudomonas* sp. LBUM223 (20ml) or *Bacillus* sp. LBUM979 (20ml) to ensure that plant growth was caused by synergy and not a dose–response relationship (Supplementary Figure 1A). The pots were kept in growth chambers (PGR15, Conviron) at 70% humidity on repetitive cycles of 18h at 28°C and 6h at 22°C (Chandra et al., 2017). The plants were watered every 2–3days as needed. Four biological replicates were used per treatment and the experiment was repeated three times for a total of 12 biological replicates per treatment. Few plants displaying aberrant morphology were eliminated from the study to exclude any variables associated with genotype. The plants were harvested 3weeks after transplantation and were dried in an oven at 70°C until completely dehydrated. The rhizosphere soil tightly bound to the roots and the bulk soil were also collected at the time of harvest, frozen in liquid nitrogen and stored at −80°C until the DNA was extracted for sequencing and marker-based 16S rRNA gene genomic analysis.

### DNA Extraction and Marker-Based Genomic Profiling

Total DNA was extracted from the rhizosphere and bulk soil (Canna coco and Promix substrate) using the Qiagen DNeasy plant mini extraction kit (Qiagen, Mississauga, Canada) for Canna coco and DNeasy PowerSoil Kit (Qiagen) for Promix. The microbial DNA from Canna coco needed to be extracted using a plant DNA extraction kit because it is a plant-based soil substrate; other soil extraction protocols do not work as efficiently (Comeau et al., 2020). Canna coco samples were disrupted in a Tissuelyser (Qiagen) at maximum speed for 6min before utilizing the DNA extraction kit. Promix soil samples were mechanically disrupted with a Fastprep (MP biomedicals) before utilizing the DNA extraction kit. The quantity of the isolated DNA was assessed with a Qubit fluorometer (Thermo Fisher, Mississauga, Canada). Subsequently, PCR amplification of the bacterial 16S rRNA gene as well as the Illumina sequencing was performed by the Centre d’Expertise et de Services Génome Québec (Montréal, Canada). The 16S rRNA gene V4 region was amplified using the primer pair 515F/806R (Caporaso et al., 2011). Purified amplicons were pooled in equimolar concentrations and paired-end sequenced (2×250) on an Illumina MiSeq platform. The raw paired-end reads were processed with the QIIME2 (version 2019.7; 2020.8) pipeline (Bolyen et al., 2019). DADA2 was used to assess the quality of the reads which included filtering, trimming, denoising, dereplicating, merging of the forward and reverse strands, as well as removing chimeras (Callahan et al., 2016). We obtained a total of 6,754,704 paired-end reads with 39,713 features identified after quality filtering. Amplicon sequence variants were aligned using MAFFT plugin which was subsequently used for the FastTree2 plugin needed for the diversity analysis (Katoh et al., 2002; Price et al., 2010). Samples used in diversity metrics were rarefied to an appropriate sampling depth of 7,063 for analysis. Alpha-diversity and statistics were calculated with the Shannon distance metric and beta-diversity was calculated using weighted UniFrac and plotted using the Vega Editor (QIIME2). Taxonomy was assigned to the 16S rRNA gene data using a Naive Bayes pre-trained Silva 132 99% OTU classifier bounded by the 515F/806R primer set (Quast et al., 2013). Differences in the abundance of bacteria was assessed using analysis of composition of microbiomes (ANCOM) and Songbird plugins from QIIME2 (Mandal et al., 2015; Morton et al., 2019). Quickly, the fit of the model for Songbird was optimized and validated by comparing to a built baseline model (Q2>0) and represented as Qurro rank plots (Fedarko et al., 2020). The predicted metagenome functions were calculated using the Phylogenetic Investigation of Communities by Reconstruction of Unobserved States (PICRUSt2) QIIME2 plugin (Segata et al., 2011; Langille et al., 2013; Douglas et al., 2020).

### Statistical Analysis

All marker-based genomic statistical tests were performed using the QIIME2 platform (Bolyen et al., 2019). More specifically, pairwise Kruskal–Wallis tests were used for assessing statistical significance of alpha-diversity (Shannon’s index) between several groups. Weighted UniFrac distance metrics were subjected to ADONIS (R2) and ANOSIM (R) statistical analysis. ANCOM or Songbird was used to assess statistical differences in taxonomy and pathway abundance between groups (Mandal et al., 2015; Morton et al., 2019). Statistical significance (ANOVA; post-hoc Dunnett) of the plant’s dry weight was assessed using GraphPad Prism 9.0.2 (GraphPad Software, Inc., San Diego, CA).

## Results

### Growth Promotion of *C. sativa* by Synergistic *Pseudomonas–Bacillus* Interaction

**Figure 1:**
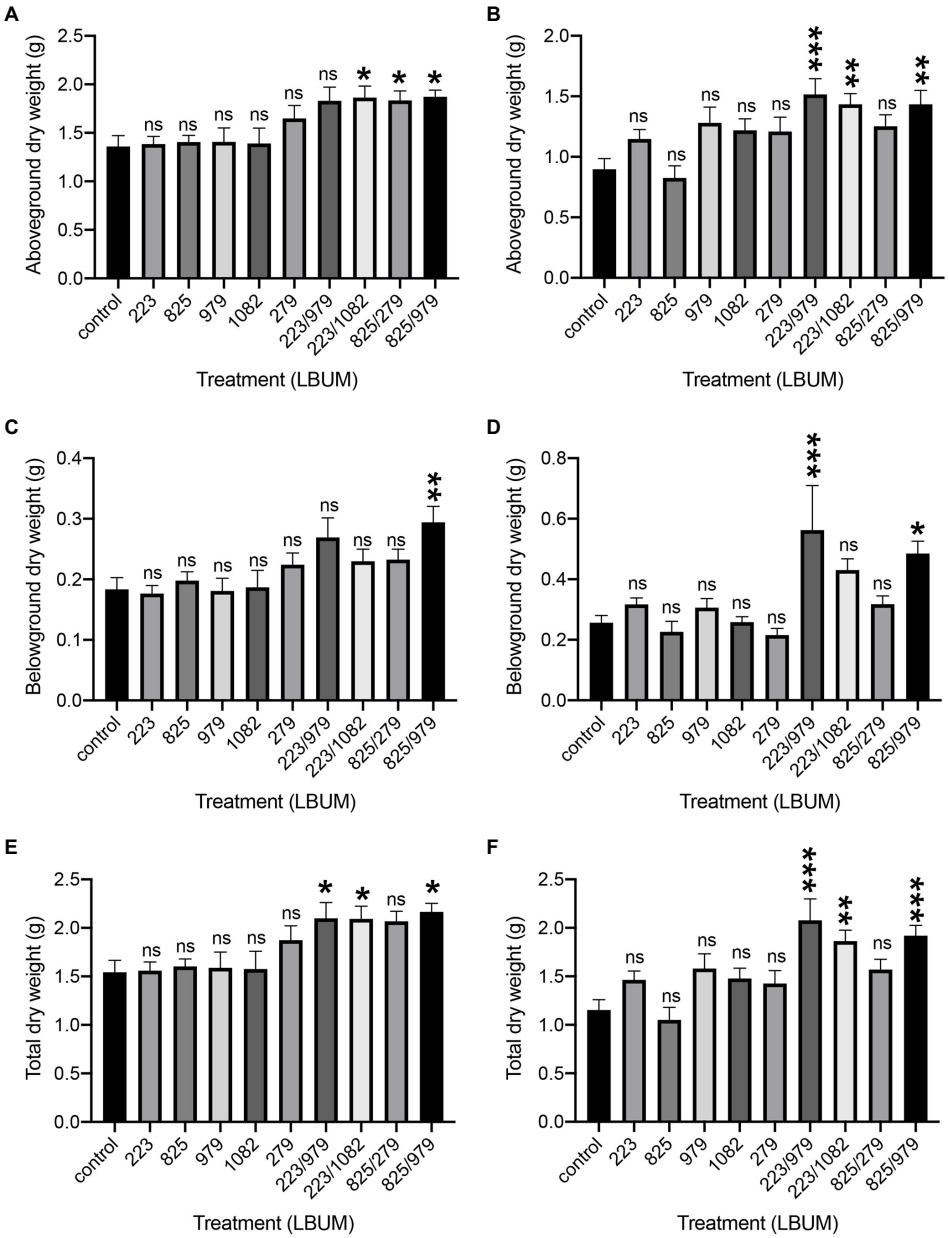
Plant growth promotion of *Cannabis sativa* treated with *Pseudomonas* spp. and *Bacillus* spp. alone or in combination, grown in Promix and Canna coco substrates. Plant growth promotion above-ground (dry weight) in **(A)** Promix and **(B)** Canna coco. Plant growth promotion below-ground (dry weight) in **(C)** Promix and **(D)** Canna coco. Total dry weight in **(E)** Promix and **(F)** Canna coco. Statistical analysis: ANOVA (^*^p<0.05, ^**^p<0.01, ^***^p<0.001, ns, not significant); mean dry weight and standard deviation of at least 12 replicates per treatment condition.

*C. sativa* was treated with two *Pseudomonas* (LBUM223 and LBUM825) and three *Bacillus* species (LBUM979, LBUM1082 and LBUM279) alone or in combination (LBUM223/979, LBUM223/1082, LBUM825/279 and LBUM825/979) to test growth promotion in Promix and Canna coco substrates (Figures 1A,C,E, 1B,D,F, respectively). While each bacterial treatment alone had no significant effect on the above-ground (shoots and leaves) or below-ground (roots) growth of *C. sativa*, some combinatorial treatments yielded significant increases in plant growth. More specifically, in Promix, combinations LBUM223/1082, LBUM825/279 and LBUM825/979 augmented the above-ground growth of *C. sativa* and LBUM825/979 had a plant-growth-promoting effect below-ground (Figures 1A,C, respectively). When looking at the total dry plant weight, combinations LBUM223/979, LBUM223/1082 and LBUM825/979 demonstrated roughly 30% increase (Figure 1E). Above-ground growth was promoted by combinations LBUM223/979, LBUM223/1082 and LBUM825/979 in Canna coco and both LBUM223/979 and LBUM825/979 had a positive effect below-ground (Figures 1B,D, respectively). In line with Promix, combinations LBUM223/979, LBUM223/1082 and LBUM825/979 significantly increased the total dry weight of the plant by up to 70% in Canna coco (Figure 1F). Furthermore, to ensure that the combinatorial treatments were not due to an increased microbial load (dose–response relationship), we inoculated double amounts of single treatment *Pseudomonas* sp. (LBUM223) or *Bacillus* sp. (LBUM979) to plants. *C. sativa* treated with a double dose of *Pseudomonas* sp. did not increase plant yield, and *Bacillus* sp. was detrimental to plant growth at double doses; in fact, *Bacillus* sp. negatively affected plant growth and vigor and no reliable measurements could be taken at the time of cultivation (Supplementary Figure 1B). Taken together, these results illustrated that the additive effect between *Bacillus* spp. and *Pseudomonas* spp. was essential to the observed plant-growth-promoting effect.

### Diversity Measurements Between Soil Type and Bacterial Inocula

**Figure 2:**
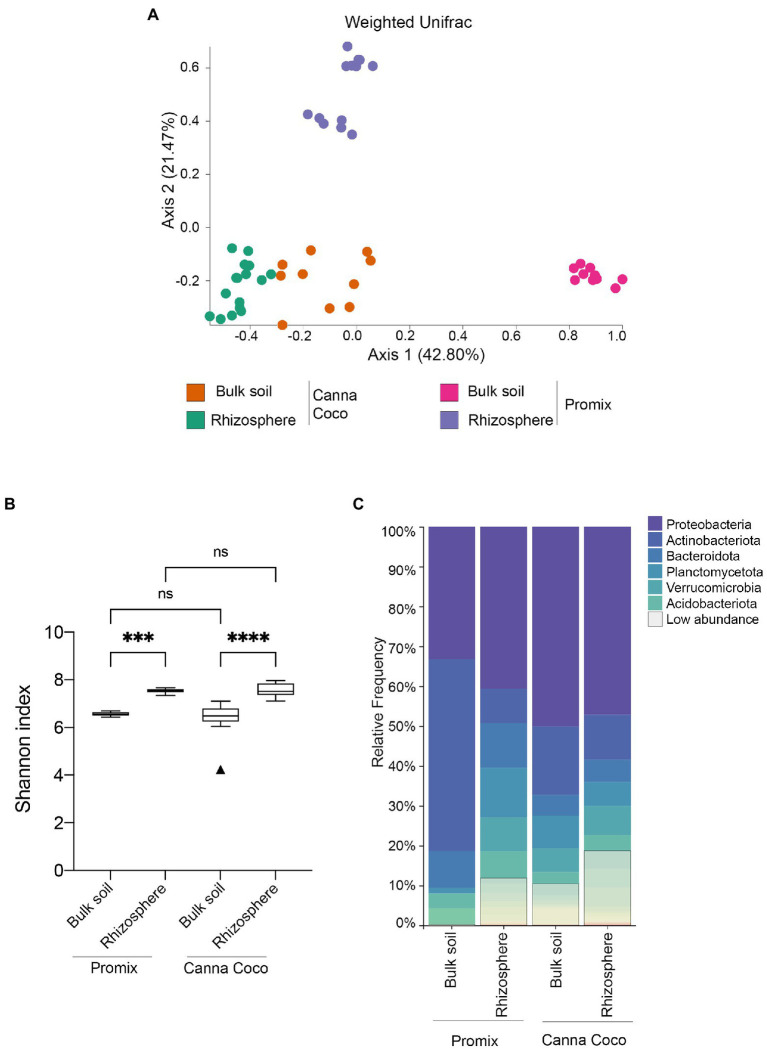
Diversity metrics and taxonomical differences, between untreated substrates, in the bacterial microbiome of *C. sativa*’s rhizosphere and bulk soil. **(A)** Weighted UniFrac PCoA plot representative of substrate differences in the bulk soil and rhizosphere. Each point represents a sample and axis 1 and axis 2 represent the percentage of variance explained by each coordinate. **(B)** Shannon’s index of variations between the bulk soil and rhizosphere of Promix and Canna coco. Kruskal–Wallis pair-wise test was used to assess statistical significance between groups (^*^p<0.05, ^**^p<0.01, ^***^p<0.001, ^****^p<0.0001). **(C)** Taxonomic bar plots of the relative frequency of bacteria at the phylum level between soil location and substrate. Low abundance taxa included: Chloroflexi, Myxococcota, Cyanobacteria, Bdellovibrionota, Gemmatimonadota, Armatimonadota, Firmicutes, Patescibacteria, Delsufobacterota, Hydrogenedentes, Dependentiae, Abditibacteriota, Sporochaetota, Fibrobacterota, Sumerlaeota, SAR324, FCPU426, Elusimicrobiota, WPS-2, RCP2-54 and MBNT15.

To functionally address the impact of the bacterial treatments on growth promotion, we extracted, sequenced and analyzed the diversity and structure of the rhizosphere and the bulk soil microbiomes from both substrates. Rarefaction curves confirmed that a sufficient sequencing quality and depth was achieved (Supplementary Figure 3). We first sought to understand native microbial differences between untreated soil types. Unsurprisingly, beta-diversity weighted UniFrac showed significant differences in microbiome composition between untreated substrates, Promix and Canna coco (bulk soils: *R*=0.920; *p*=0.001, rhizospheres *R*=0.998; *p*=0.001; Figure 2A). More interestingly, the microbial structure between the bulk soil and rhizosphere of Promix presented a greater shift than that of Canna coco (*R*=1, *p*=0.001; *R*=0.732, *p*=0.001, respectively; Figure 2A). The alpha–diversity measured by the Shannon index, taking into account both the richness and evenness of the community, was increased in the rhizosphere when compared to bulk soil albeit showed no differences between substrates (Figure 2B; Comeau et al., 2020). Furthermore, the bacteria that inhabit the bulk soil and rhizosphere had notable varying frequencies between substrate and location (Figure 2C). Taken together, the native taxonomical differences and phylogenetic diversity (weighted UniFrac) between substrates intimated the idea that the combinatorial plant-growth-promoting treatments, which are found in both evidently different substrates, are not only flexible but robust to diverse native microbial compositions.

**Figure 3:**
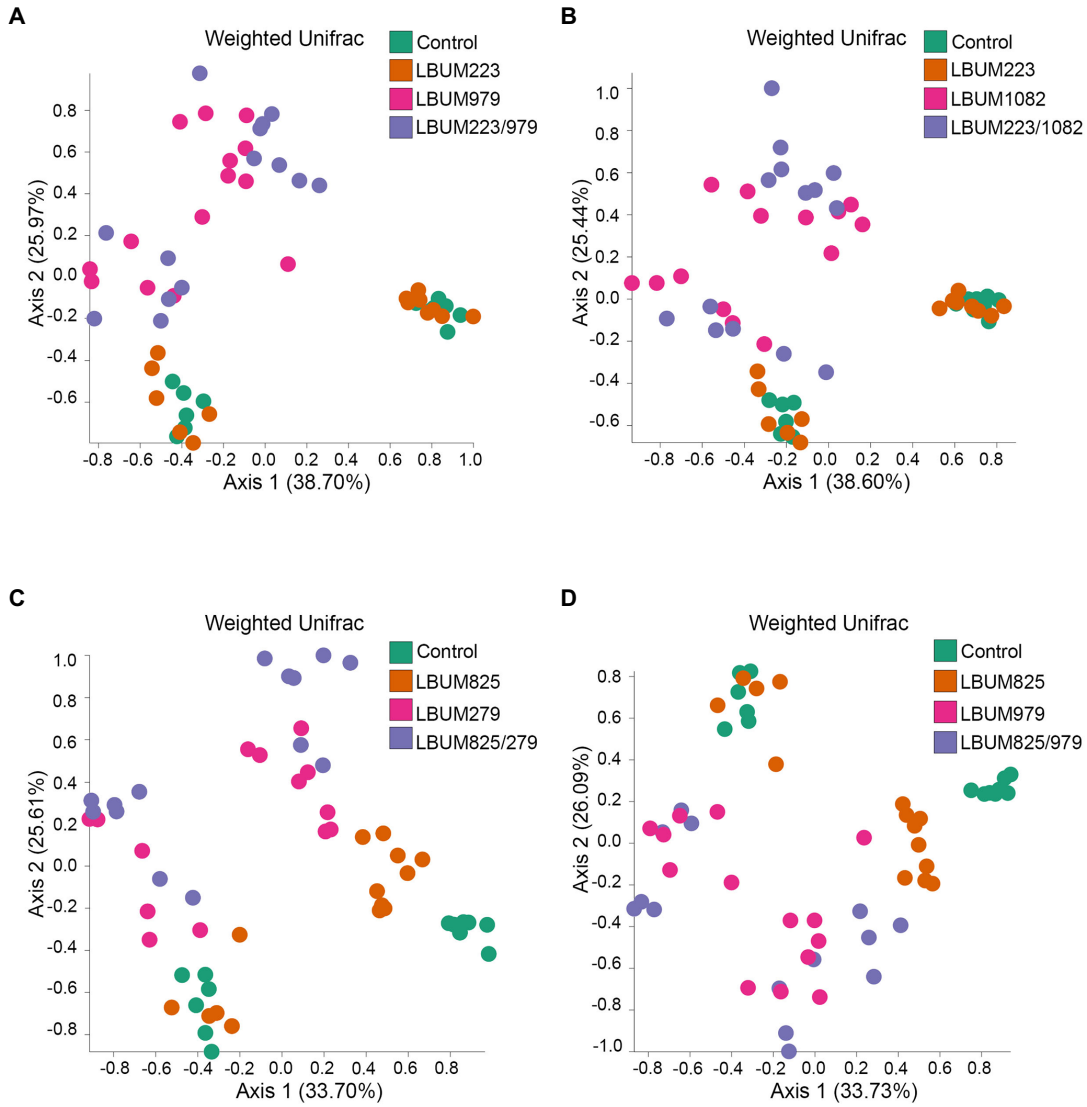
Weighted UniFrac beta-diversity metric between treatment conditions in Promix rhizosphere. PCoA plot representative of beta-diversity between treatment groups; **(A)** LBUM223 and LBUM979, **(B)** LBUM223 and LBUM1082, **(C)** LBUM825 and LBUM279 **(D)** LBUM825 and LBUM979, alone or in combination (the control refers to plants inoculated with water). *Pseudomonas* spp. treatments are represented by orange circles, *Bacillus* spp. by pink circles, combinatorial treatments by purple circles and the controls in green. Each point represents a sample and axis 1 and axis 2 represent the percentage of variance explained by each coordinate. Statistical analysis is found in the text (ANOSIM and ADONIS).

**Figure 4:**
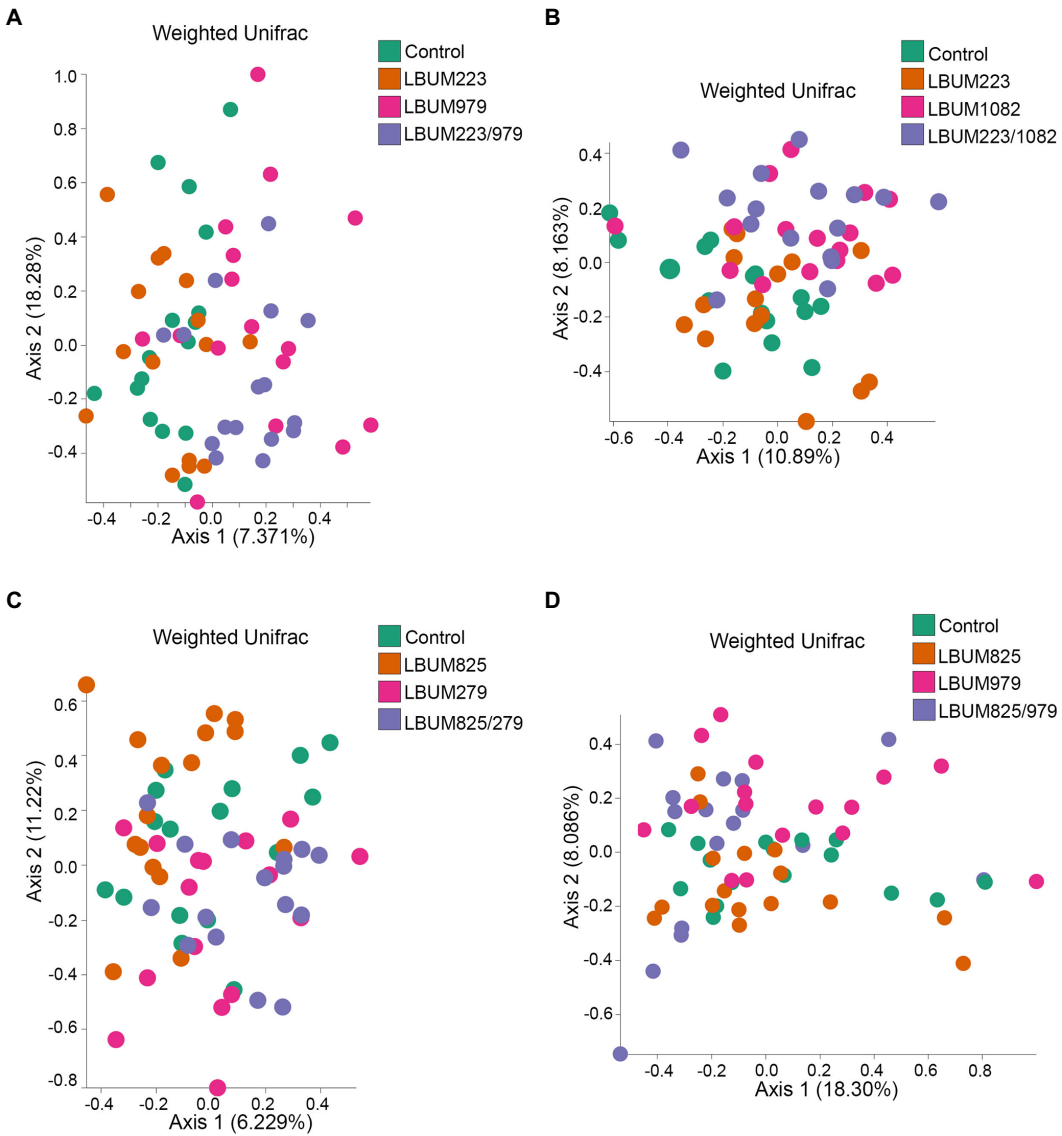
Weighted UniFrac beta-diversity metric between treatment conditions in Canna coco rhizosphere. PCoA plot representative of beta-diversity between the treatment groups; **(A)** LBUM223 and LBUM979, **(B)** LBUM223 and LBUM1082, **(C)** LBUM825 and LBUM279 **(D)** LBUM825 and LBUM979, alone or in combination (the control refers to plants inoculated with water). *Pseudomonas* spp. treatments are represented by orange circles, *Bacillus* spp. by pink circles, combinatorial treatments by purple circles and the control by green. Each point represents a sample and axis 1 and axis 2 represent the percentage of variance explained by each coordinate. Statistical analysis is described in the text (ANOSIM and ADONIS).

Next, we utilized the marker-based genomic information from the rhizosphere to uncover differences between treatments that promote plant growth and those that do not. The treatment groups showing above-ground or below-ground growth promotion in Promix and Canna coco were analysed for changes in beta-diversity using weighted UniFrac (Figures 3, 4, respectively). Alpha-diversity Shannon index was also measured but no changes were identified between treatment groups in the Promix and Canna coco substrates (Supplementary Figure 2A–D and 1E–H, respectively). All combinatorial treatments in Promix, namely LBUM223/979, LBUM223/1082, LBUM825/279 and LBUM825/979, clustered away from the untreated control (*R*=0.510, *p*=0.001; *R*=0.504, *p*=0.001; *R*=0.677, *p*=0.001; *R*=0.597, *p*=0.001, respectively), and this clustering could partially be explained by the bacterial treatment (*R*^2^=0.280, *p*=0.001; *R*^2^=0.304, *p*=0.001; *R*^2^=0.288, *p*=0.001, respectively). Furthermore, treatment groups LBUM223/979 and LBUM223/1082 presented clustering on PCoA plot between *Pseudomonas* LBUM223 and untreated controls while *Bacillus* LBUM979 and LBUM1082 clustered with combinatorial treatments, highlighting *Bacillus* spp. as the main modulator of the microbiome’s diversity in Promix (Figures 3A,B, respectively). This, however, was not the case for combinatorial treatments LBUM825/279 and LBUM825/979 (Figures 3C,D). In combinatorial treatment LBUM825/279, *Pseudomonas* LBUM825 and the water control still remained clustered together; however, *Bacillus* LBUM279 clustered slightly away from the combinatorial treatment (*R*=0.134, *p*=0.027; Figure 3C). This tendency was then reversed in combinatorial treatment LBUM825/979; *Pseudomonas* LBUM825 clustered slightly away from the water control (*R*=0.119, *p*=0.049) while LBUM979 remained grouped with the combinatorial treatment (Figure 3D). To corroborate these findings, diversity measurements using Bray–Curtis dissimilarity were also calculated. As demonstrated using weighted UniFrac, combination treatments clustered away from water control, *Pseudomonas* spp. clustered with the water control, and *Bacillus* spp. clustered away from the water control. Furthermore, *Bacillus* LBUM1082 clustered with its respective combination treatment and *Bacillus* LBUM279 and LBUM979 clustered away from their respective combination treatments (Supplementary Figure 4).

As seen in the Promix substrate, the bacterial treatments in Canna coco presented similar clustering patterns, albeit with less discernable shifts on PCoA plots (Figure 4). Combinations LBUM223/979, LBUM223/1082 and LBUM825/279 caused a shift in beta-diversity away from the water control (*R*=0.169, *p*=0.002; *R*=0.187, *p*=0.003; *R*=0.128, *p*=0.003, respectively) and this clustering could also be partly explained by the bacterial treatments (*R*^2^=0.087, *p*=0.004; *R*^2^=0.092, *p*=0.002; *R*^2^=0.02, *p*=0.001) (Figures 4A–C). Moreover, in combinations LBUM223/979, LBUM223/1082 and LBUM825/279, *Pseudomonas* LBUM223 and LBUM825 clustered with the water control and *Bacillus* LBUM979, LBUM1082 and LBUM279 clustered with their respective combinatorial treatment (Figures 4A–C). Although the combination LBUM825/979 demonstrated growth promotion in *C. sativa*, changes in microbiome beta-diversity in Canna coco was marginal, showing only *Bacillus* LBUM979 as clustering away from the water control (*R*=0.145, *p*=0.009; Figure 4D). Bray-Curtis diversity measurement in Canna coco were not as conclusive as with weighted UniFrac. Although shifts in diversity in this substrate remained marginal, differential clustering was clearly observed between *Bacillus* spp. and all other combinations, that is to say, *Bacillus* spp. likely caused the greatest shift in diversity (Supplementary Figure 5). Altogether, beta-diversity measurements indicated that *Bacillus* spp. was driving changes in beta-diversity and, although not having had a substantial effect on beta-diversity, *Pseudomonas* spp. were essential to the observed growth promotion of *C. sativa* (Figure 1). We thus hypothesized that *Bacillus* spp. and *Pseudomonas* spp. in combinatorial treatments may have had a positive effected on the native bacteria, or on each other, which in turn, could have had a plant-growth-promoting effect. To investigate this further, we sought to identify taxonomical differences between treatment groups.

### Identification of Taxonomic Differences in the Rhizosphere Between Bacterial Inocula

**Figure 5:**
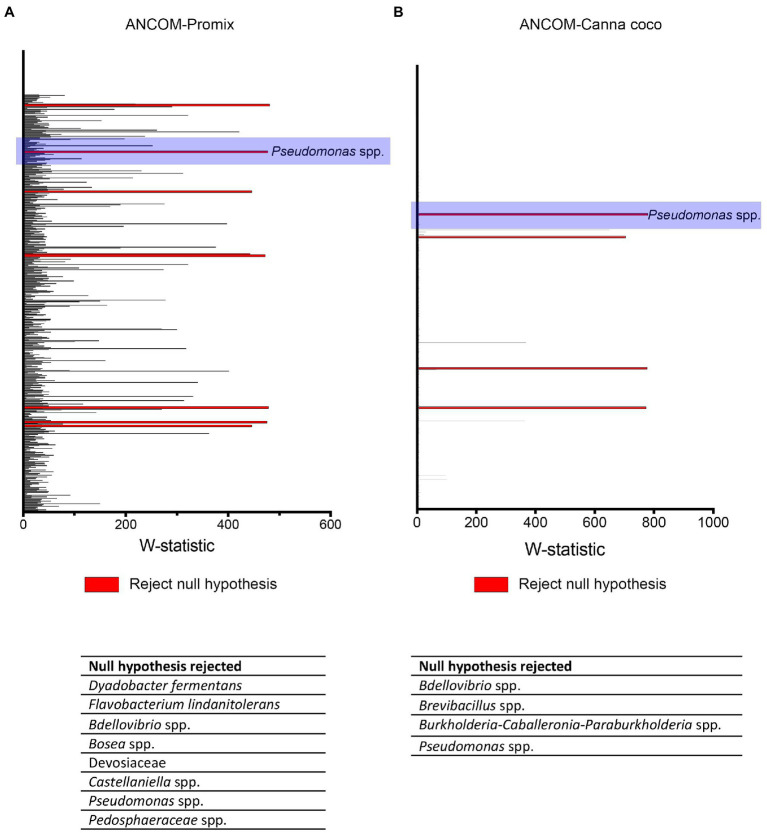
ANCOM analysis of statistically important taxonomical differences between treatment groups. ANCOM output in **(A)** Promix and **(B)** Canna coco. Bacteria identified as differentially abundant (rejecting the null hypothesis) are listed at the bottom of each graph.

To better understand the interaction between *Bacillus* spp., *Pseudomonas* spp. and *C. sativa*, we utilized differential abundance measures to identify bacteria responsible for the shifts in beta-diversity and those associated with plant growth promotion. The taxonomical differences responsible for the shift in diversity were brought to light using the stringent statistical framework ANCOM (Figure 5; Mandal et al., 2015).

**Figure 6:**
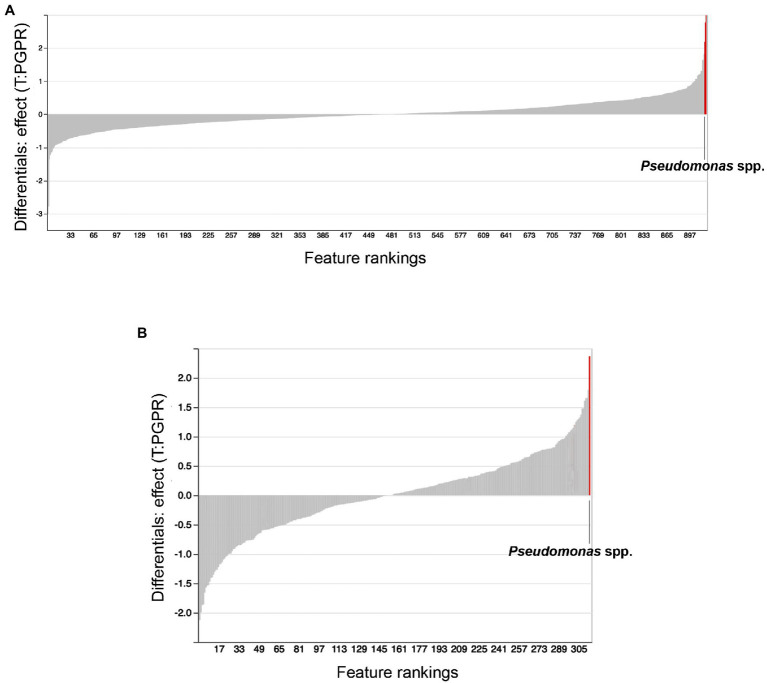
Songbird feature rankings of bacteria to plant growth promotion. Songbird differentials ranked in Qurro plot for substrates **(A)** Promix and **(B)** Canna coco. Highest ranked bacteria are highlighted in red and identified in the plots.

We used ANCOM to identify differential abundances between treatments, that is to say, between the control (water treatment), *Bacillus* spp., *Pseudomonas* spp. and combinatorial treatments. Furthermore, we sought to find taxa which were differentially abundant in both substrates. Our reasoning was that, if the same taxonomical differences were found in both substrates, they would have a greater chance of being vital to shifts in beta-diversity and plant growth promotion. Along with other bacteria, *Pseudomonas* spp. were identified as differentially abundant between treatments in Promix (Figure 5A). However, only *Pseudomonas* spp. and *Bdellovibrio* spp. were found to be differentially abundant in both substrates (Figures 5A,B). Notably, the addition of treatments in Promix had a greater effect on the inhabitants of the rhizosphere than in Canna coco; nevertheless, the plant-growth-promoting effect was observed in both substrates (Figures 5A,B). Songbird also estimates differential abundance by using reference frames that negate the need for measuring total microbial load or microorganism count while diminishing false discovery rates, even more so when compared to ANCOM (Morton et al., 2019). Using Qurro rank plots, taxonomic features are sorted as being weakly (far left) or strongly (far right) associated with a covariate (Fedarko et al., 2020). Here, Songbird differentials ranked *Pseudomonas* spp. as being amongst the bacteria most strongly associated with plant-growth-promoting combinatorial treatments in Promix (Figure 6A). Other bacteria identified using ANCOM, including *Bdellovibrio* spp., were not evidently associated with plant growth promotion. Using Songbird, changes in Canna coco substrate, as with ANCOM, were negligible when compared to Promix, and so it was difficult to associate with great certainty a treatment outcome to a covariate (Q2 nearing 0). Nevertheless, the bacteria most highly ranked, and thus most associated with plant growth promotion in Canna coco, was *Pseudomonas* spp., as with Promix (Figure 6B). Although Songbird statistics were weak in Canna coco substrate, we do not expect main conclusions to be disputed when considering orthogonal methods used, i.e. ANCOM and Songbird statistics from both substrate types. Surprisingly, *Bacillus* spp. was not identified in the ANCOM or Songbird analyses. Altogether, ANCOM and Songbird hinted towards the importance of *Pseudomonas* spp. in plant-growth-promoting combinatorial treatments. However, because there was no species level identification, it was impossible to differentiate native *Pseudomonas* spp. from *Pseudomonas* spp. used as treatments. Although we could not differentiate *Pseudomonas* spp. at the species level, we sought to elucidate predicted metabolic pathways responsible for plant growth promotion.

### Predicted Pathway Abundance in PGPR Treatments

**Figure 7:**
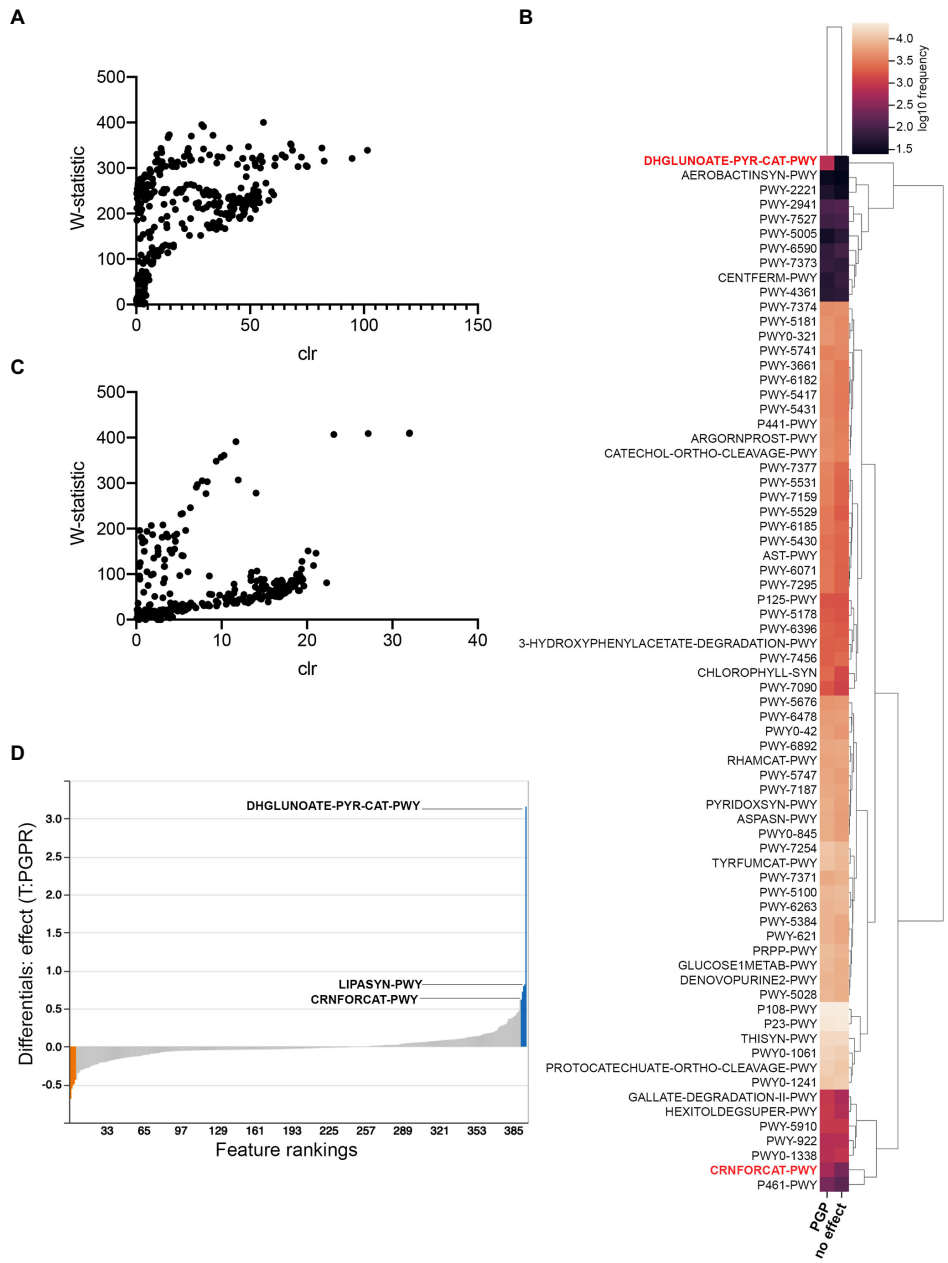
PICRUSt2 predicted pathway variations in plant-growth-promoting treatments compared to those with no effect. **(A)** ANCOM analysis in Promix **(B)** Heatmap of ANCOM output in Promix of relative abundance of pathways between treatments having a plant-growth-promoting effect and no effect (pathways in red have been identified by both ANCOM and Songbird) **(C)** ANCOM analysis in Canna coco **(D)** Songbird analysis in Promix. In blue are the top pathways associated with plant growth promotion and in orange the pathways least associated with this feature.

To identify possible metabolic pathways linked to PGPR in *C. sativa*, we utilized the analytic pipeline PICRUSt2 (Langille et al., 2013; Douglas et al., 2020; Figure 7). This tool helps predict pathways differentially abundant between treatment groups, herein, between treatment groups having plant-growth-promoting effects (combinatorial treatments LBUM223/979, LBUM223/1082, LBUM825/279 and LBUM825/979) and those that are neutral (single inoculants). ANCOM discerned 71 differently abundant MetaCyc pathways in Promix, schematically represented by heatmaps (Figures 7A,B, respectively). Overall, pathways were more often predicted to be more abundant in plant-growth-promoting combinatorial treatments; the pathways identified included many catabolic pathways necessary for life in the rhizosphere such as sugar, amino acid and aromatic compound metabolism, as well as the biosynthesis of plant mediators (Figure 7B). Notably, ANCOM identified a greater abundance of pathways in Promix than in Canna coco substrate (Figures 7A,C, respectively). In practical terms, pathways that have a greater W-statistic and centred log ratio are likely to reject the null hypothesis; this shift upwards and to the right was evidentially more pronounced in Promix than in Canna coco (Figures 7A,C, respectively). In fact, only five pathways could be identified as differently abundant in Canna coco, all of which were also found in Promix: DHGLUCONATE-PYR-CAT-PWY (oxidative glucose degradation), CRNFORCAT-PWY (creatinine degradation I), PWY-2941 (L-lysine biosynthesis II), PWY-4361 (*S*-methyl-5-thio-α-D-ribose 1-phosphate degradation I) and PWY-7527 (L-methionine salvage cycle III). These five pathways could easily be linked back to either *Pseudomonas* spp. or *Bacillus* spp. metabolism. The DHGLUCONATE-PYR-CAT-PWY (oxidative glucose degradation) metabolic pathway, which was predicted to be relatively more abundant in plant-growth-promoting treatments, has been documented in *Pseudomonas* spp., including the known PGPR *P. fluorescens* (Fuhrer et al., 2005). Likewise, CRNFORCAT-PWY (creatinine degradation I), a pathway found in *Pseudomonas* spp., although not necessarily linked to PGPR metabolism, was also predicted to be more abundant (Shimizu et al., 1989). Metacyc identifiers PWY-2941 (L-lysine biosynthesis II), PWY-4361 (*S*-methyl-5-thio-α-D-ribose 1-phosphate degradation I) and PWY-7527 (L-methionine salvage cycle III) are pathways commonly found in Firmicutes such as *Bacillus* spp. and were all predicted to be less abundant in plant growth promotion treatments. Amongst these pathways common between substrates, DHGLUCONATE-PYR-CAT-PWY (oxidative glucose degradation) and CRNFORCAT-PWY (creatinine degradation I) were also highly ranked by Songbird in Promix (Figure 7D). More likely, creatine metabolism is only linked to *Pseudomonas* spp. and not likely to plant growth promotion. As previously described, sugar metabolism, such as DHGLUCONATE-PYR-CAT-PWY (oxidative glucose degradation) is a property common to plant associated bacteria in general but has also been documented in the *C. sativa* microbiome (Levy et al., 2017; Comeau et al., 2020). Phosphorus solubilization by PGPR has been associated to sugar metabolism which produces acids capable of solubilizing this essential element and making it bioavailable to plants (Rodríguez and Fraga, 1999; Goswami et al., 2014). Conversely, the other three pathways identified by ANCOM; PWY-2941 (L-lysine biosynthesis II), PWY-4361 (*S*-methyl-5-thio-α-D-ribose 1-phosphate degradation I) and PWY-7527 (L-methionine salvage cycle III) were not ranked as associated with plant growth promotion. Additional pathways not discerned by ANCOM were highly ranked in the Songbird analysis, namely the LIPASYN-PWY (phospholipases) pathway. All in all, most pathways identified had clear lines of evidence pointing towards typical *Pseudomonas* and *Bacillus* PGPR metabolic pathways and colonization traits.

## Discussion

We have identified *C. sativa*-growth-promoting consortia consisting of specific *Pseudomonas* spp. isolates and specific *Bacillus* spp. isolates. The analysis of marker-based Illumina sequencing from extracted rhizosphere DNA suggested that *Bacillus* spp. caused the greatest measurable shift in beta-diversity and that *Pseudomonas* spp., but not *Bacillus* spp., was associated to the plant-growth-promoting effect. As more molecular insight and interdisciplinary tools would be needed to build inter-species networks of interactions, we can only infer plausible methods of interactions that could lead to growth promotion. For example, a linear interaction between *Bacillus* spp., *Pseudomonas* spp. and *C. sativa* could have been possible, where *Bacillus* spp. is a key contributor to *Pseudomonas* spp. colonization, potentializing an interaction between the latter and *C. sativa*. It is also plausible that instead of a schematic linear interaction, *Bacillus* spp. could directly interact with the root system of *C. sativa*, mediating a systemic root response such as the systematically induced root exudation of metabolites, favorable to the colonization of other species in the rhizosphere microbiome (Korenblum et al., 2020). Alternatively, interactions between *Bacillus* spp. and *Pseudomonas* spp. might have favored or hindered the proliferation of native bacteria, fungi or oomycetes, amongst others, which then had a positive effect on *C. sativa* growth. Furthermore, as *Bacillus* spp. was not identified as differently abundant between treatments in the ANCOM or Songbird analyses, it is possible that these bacteria modulate the rhizosphere microbiome but do not colonize over long-term periods. The direct quantification of *Pseudomonas* spp. and *Bacillus* spp. colonization over time would help in delineating these hypotheses. Greater molecular insight into this tripartite interaction is needed to better understand the events leading to plant growth promotion.

Manipulating the agroecosystem remains a formidable task owing to the complexity and interconnectedness between countless microorganisms and the host (Berendsen et al., 2012; Agler et al., 2016; Busby et al., 2017). Although this study focused solely on the bacterial microbiome, understanding the fungal, viral and archaeal microbiomes, and how they interact with one another and with their host, is essential to understanding the microbiome as a whole and remains a formidable challenge. In this study, predictive pathway analysis identified several known PGPR pathways possibly responsible for inter-species interaction and plant growth promotion, notably, sugar metabolism, protocatechuate and aromatic compounds degradation, 2,3-butanediol biosynthesis, amongst others. Phospholipase metabolic pathway identified in the study is also an important secondary messenger pathway in plants, regulating response to phytohormones and pathogen elicitation (Ryu, 2004). In addition, a great deal of phosphorus in the soil is bound to biomolecules including nucleic acids, phosphorylated proteins and phospholipids. Secreted bacterial phospholipases have the capacity to liberate phosphorus from phospholipids, which the plant could assimilate, potentially augmenting plant growth (Martínez et al., 2012; Tapia-Torres et al., 2016). The phospholipase pathway together with sugar metabolism in *Pseudomonas* spp. might significantly increase the availability of essential soil nutrients for the plant and promote plant growth. An increase in the plant sugar content for example, following inoculation with PGPR, has been previously associated with increased plant stress tolerance (Kang et al., 2019; Khan et al., 2019). This might have contributed, amongst others, to plant biomass accumulation under our conditions. Metabolomics studies would be helpful in this context to better characterize changes in plant metabolite profiles that directly contributed to the observed plant growth increase. Not limiting our analysis to pathways common between substrates, we found many other pathways highlighted by ANCOM that have been previously associated to *Pseudomonas* spp. and *Bacillus* spp. PGPR. To name a few, the PROTOCATECHUATE-ORTHO-CLEAVAGE-PWY pathway (protocatechuate degradation II) is habitually found in plant-growth-promoting microorganisms, including PGPR pseudomonads (Harwood and Parales, 1996; Shen et al., 2013). Pathways PW5431 (aromatic compounds degradation *via* β-ketoadipate) and 3-HYDROXYPHENYLACETATE-DEGRADATION-PWY (4-hydroxyphenylacetate degradation) were also identified in a comparative genomic study of plant-growth-promoting pseudomonads (Li et al., 2010; Shen et al., 2013). The production of an important PGPR volatile compound, 2,3-butanediol, by *Bacillus* spp. superpathways PWY-6396 (superpathway of 2,3-butanediol biosynthesis) and P125-PWY [superpathway of (R,R)-butanediol biosynthesis], has also been associated with plant growth promotion, induced systemic resistance and more recently to the ability to modulate rhizosphere bacteria colonization (Ryu et al., 2004; Hahm et al., 2012; Yi et al., 2016). Given the limitations of marker-based sequencing in identifying metabolic pathway activation, we are mindful not to overly infer. As these are only predictions, the importance of these pathways should be addressed in the bacteria surveyed by reverse genetics and by targeted metabolic studies.

Intriguingly, the combination LBUM825/LBUM279 only demonstrated above-ground growth promotion in Promix and none in Canna coco, presenting contrariety in this treatment combination between substrates, possibly because of soil type dependencies linked to this treatment combination. As previously demonstrated, soil physicochemical properties have a considerable effect on the overall health of the plant but also on the soil microbiome, which may explain the observed soil type dependencies linked to this treatment combination (Fierer and Jackson, 2006; Leite et al., 2017). The lack of efficacy for combinatorial treatment LBUM825/LBUM279 in Canna coco, and a generally higher standard deviation for all treatment groups in this substrate might also be due to the required addition of fertilizers which would mask the PGPR effect in nutrient enriched conditions. Despite its usefulness in the present study for validating taxonomical finds in Promix, Canna coco demonstrated very weak fluctuations in the diversity and structure of the microbiome. Although this substrate is used commercially in the cultivation of cannabis, the lack of changes in these features may be indicative of the drawback for using this substrate, possibly hindering the root system to call to specific microbes under varying conditions, namely under stress (Fierer, 2017).

Under the conditions used in this study, we cannot completely rule out the possibility that some microbial metabolites could have been added during inoculation as part of the culture medium. These metabolites might have growth-promoting effects and/or effects on the resident bacteria. These effects, if any, could also potentially have been modulated by the different soil substrates used under our conditions. However, the fact that the experiments were performed over many weeks and that proper controls using double inoculation doses did not yield increased plant-growth-promoting effects suggest that these metabolites did not have a significant impact on the main results presented in thus study.

A common issue with the commercialization of PGPR remains the lack of reproducibility and consistency in practice (Girlanda et al., 2001; Walsh et al., 2003; Castro-Sowinski et al., 2007). To better understand growth promotion, more mechanistic insight, and description of the effect of the treatments on the microbiome as a whole are needed. More precisely, describing how the diversity, structure and function of the microbiome has changed post-treatment with PGPR could avoid pitfalls in practice (Lozupone et al., 2012; Dessaux et al., 2016; Hartman et al., 2018; Toju et al., 2018). Our work creates an essential base that will facilitate further work in acquiring deeper molecular insight into the interactions between bacterial inhabitants and their host, which could enable a more tailored matching and engineering of PGPR to *C. sativa*, in lieu of arduous screening methodologies.

## Data Availability Statement

The datasets presented in this study can be found in online repositories. The names of the repository/repositories and accession number(s) can be found at: www.ncbi.nlm.nih.gov/bioproject/, PRJNA702927.

## Author Contributions

DC carried out all experiments, part of the sequencing analysis (Figures 1–5) and writing of the manuscript. AN and CB are responsible for designing the plant growth promotion experiments, choosing the bacteria to be surveyed and editing the manuscript. NB is responsible for part of the sequencing analysis (Figures 6, 7) and editing the manuscript. MF and DJ supervised and conceived the project and edited the manuscript. All authors contributed to the article and approved the submitted version.

## Funding

This project was funded by a collaborative Genome Canada/Atlantic Canada Opportunity Agency/New Brunswick Innovation Foundation grant awarded to MF and DJ.

## Conflict of Interest

The authors declare that the research was conducted in the absence of any commercial or financial relationships that could be construed as a potential conflict of interest.

## Publisher’s Note

All claims expressed in this article are solely those of the authors and do not necessarily represent those of their affiliated organizations, or those of the publisher, the editors and the reviewers. Any product that may be evaluated in this article, or claim that may be made by its manufacturer, is not guaranteed or endorsed by the publisher.
