# Supplementary figures and images for: Interactions Between Bacillus Spp., Pseudomonas Spp. and Cannabis sativa Promote Plant Growth
Source: Front Microbiol. 2021 Sep 20;12:715758. doi: 10.3389/fmicb.2021.715758 (PMC8488376; doi:10.3389/fmicb.2021.715758)

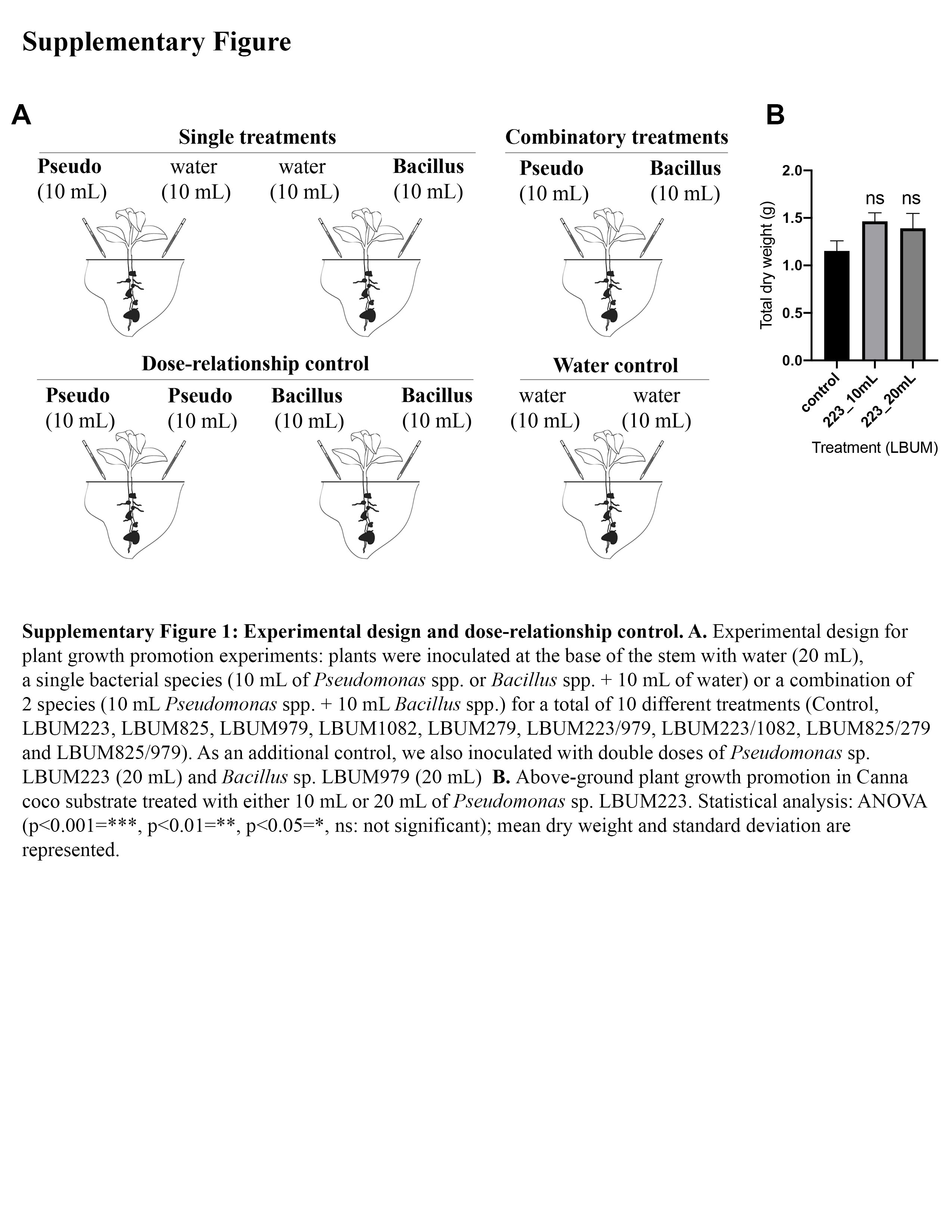

Supplement: Supplementary file 1 [file Image_1.JPEG]

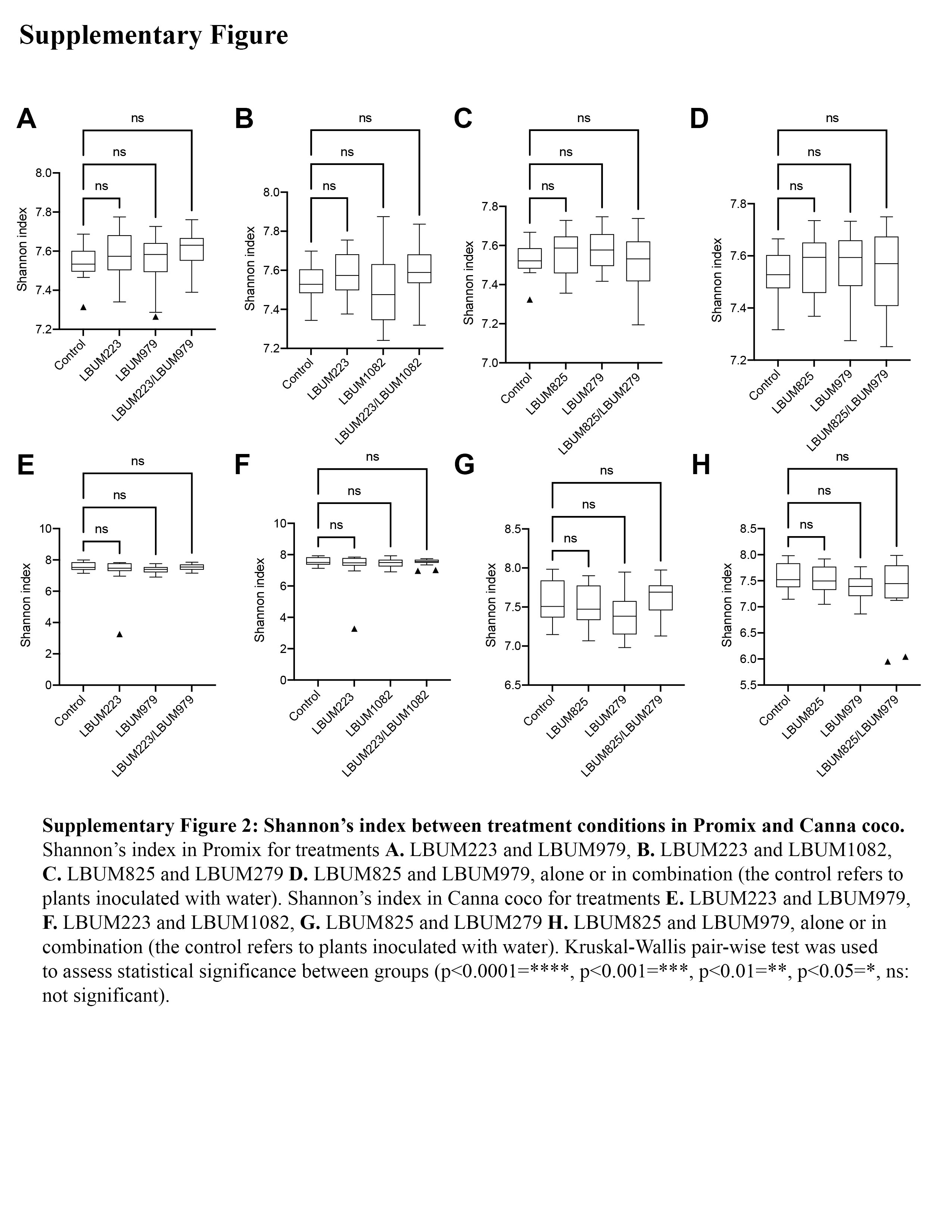

Supplement: Supplementary file 2 [file Image_2.JPEG]

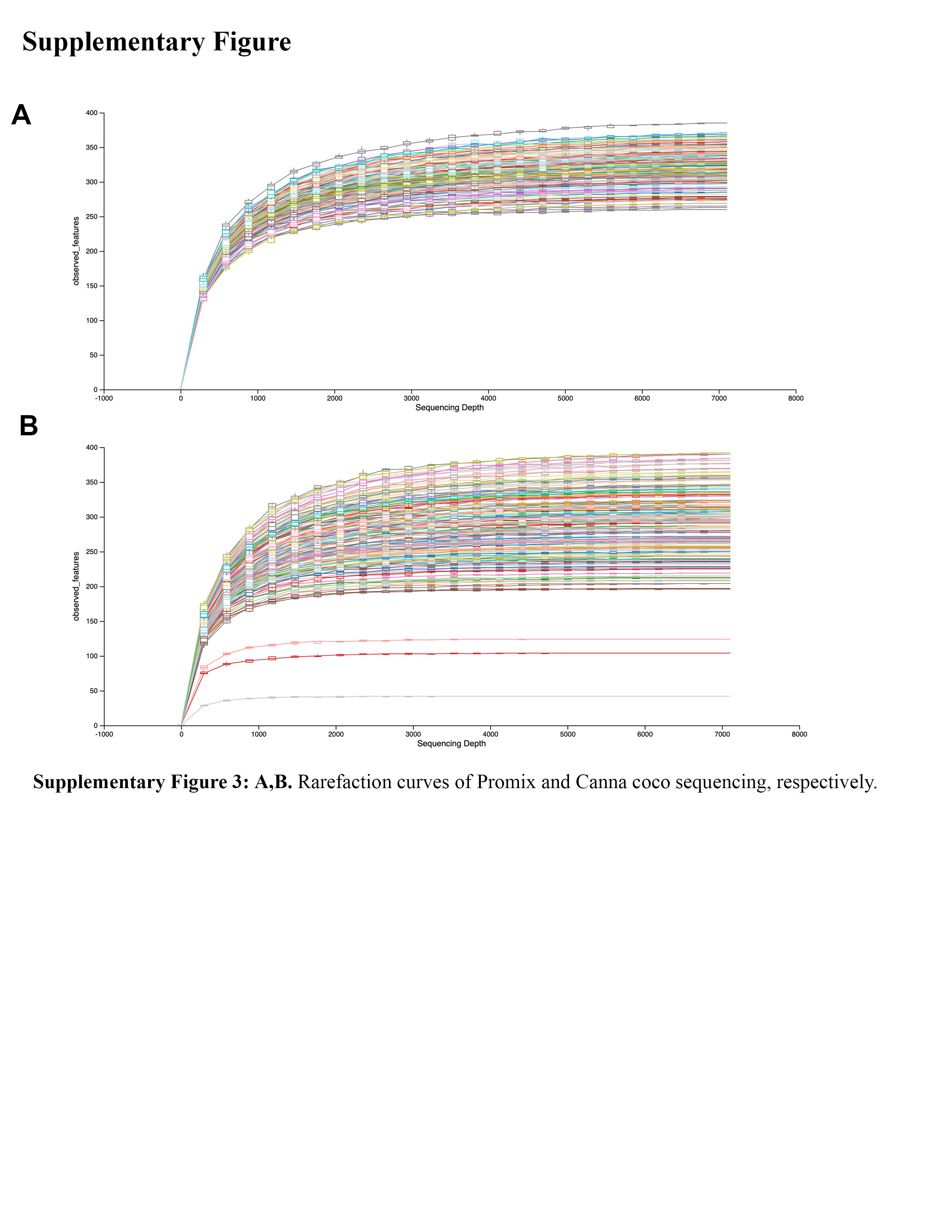

Supplement: Supplementary file 3 [file Image_3.JPEG]

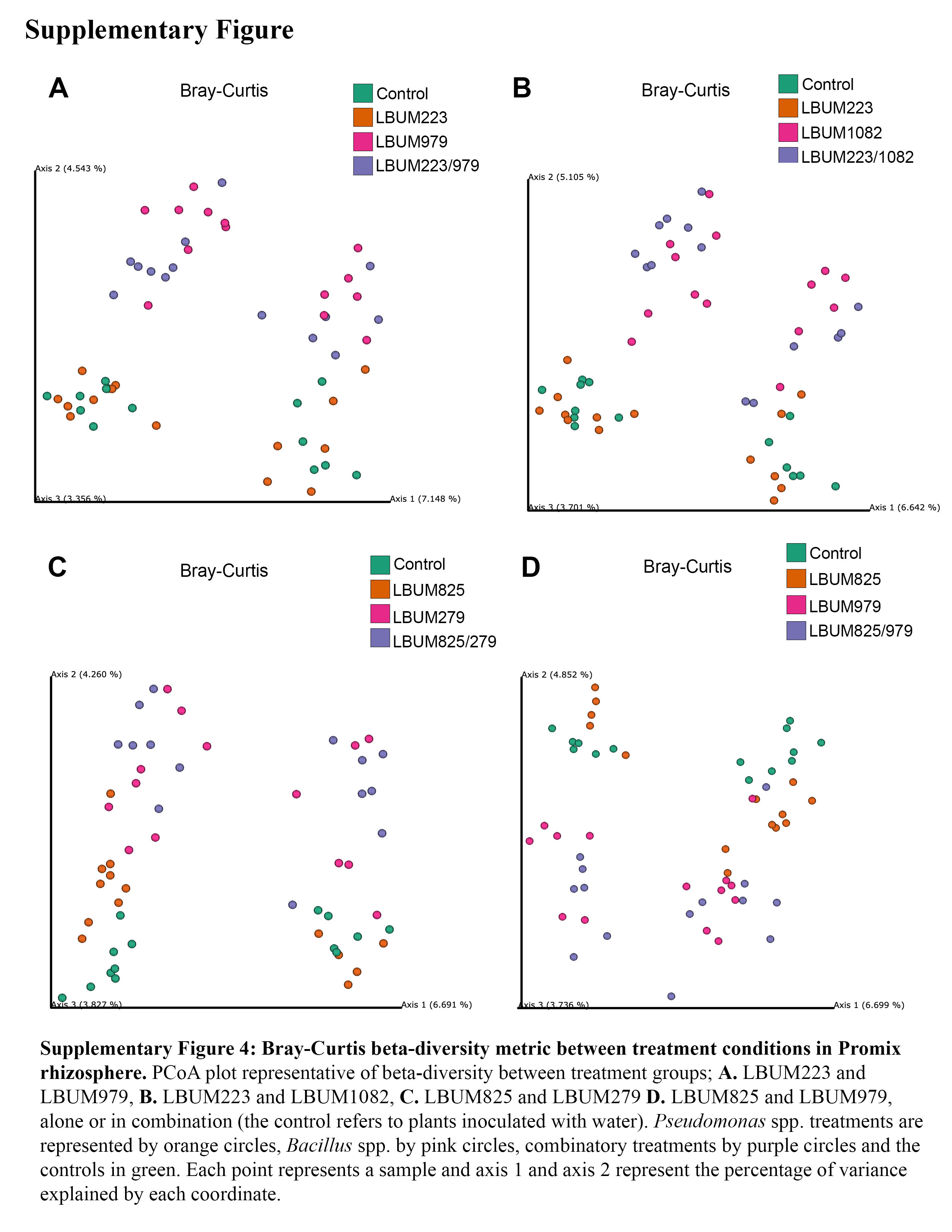

Supplement: Supplementary file 4 [file Image_4.JPEG]

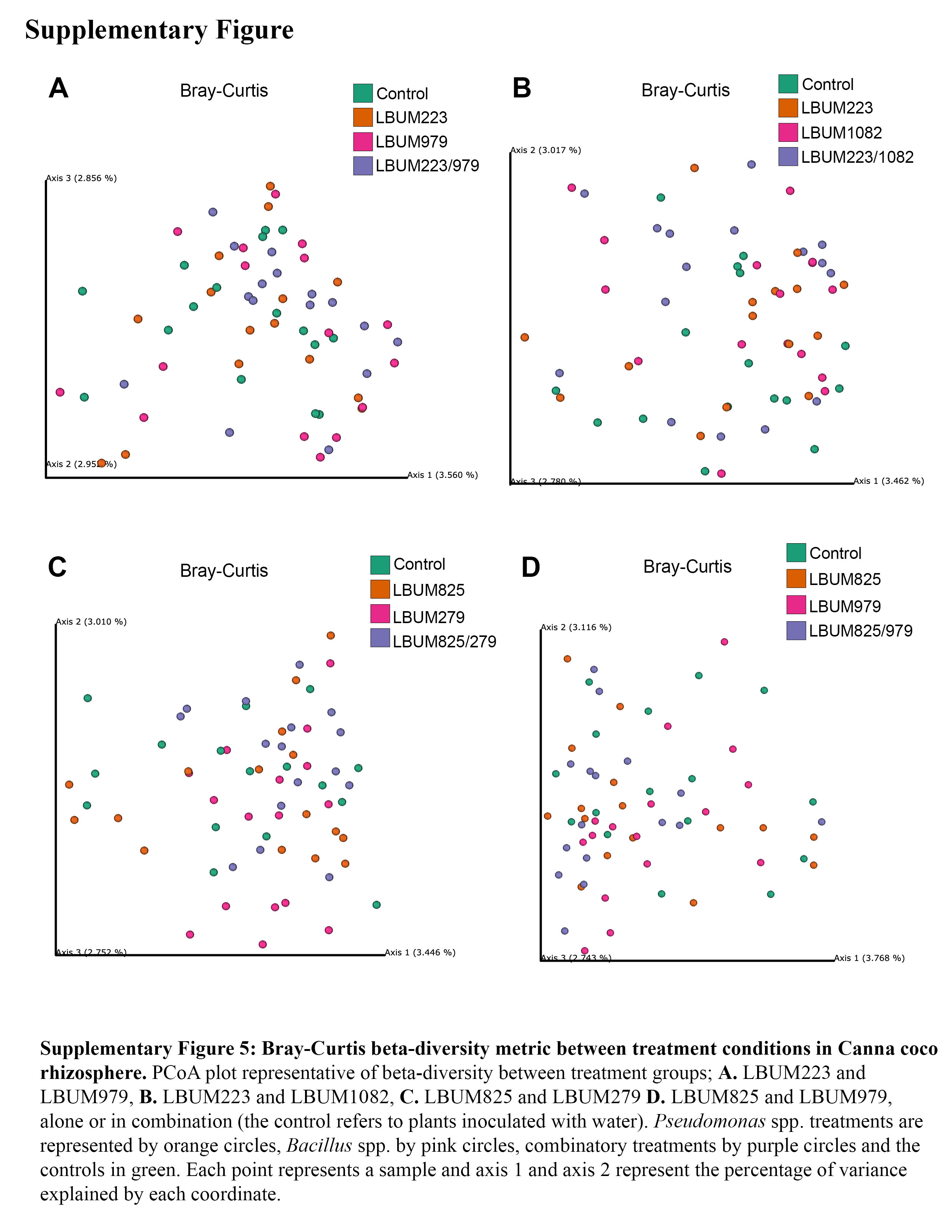

Supplement: Supplementary file 5 [file Image_5.JPEG]
